# Characteristic metabolite profile of 10 colorectal cancer-related bacteria

**DOI:** 10.3389/fonc.2025.1604876

**Published:** 2025-07-14

**Authors:** Yongqiang Liu, Wangli Mei, Xinyan Huang, Xudong Yao, Cheng Kong, Yifan Chen

**Affiliations:** ^1^ Department of Urology, Shanghai Tenth People’s Hospital, School of Medicine, Tongji University, Shanghai, China; ^2^ Department of Urology, Shanghai East Hospital, School of Medicine, Tongji University, Shanghai, China; ^3^ Anesthesia and Surgery Department, Nanjing Drum Tower Hospital Affiliated to Nanjing University, Nanjing, China; ^4^ Department of Oncology, Shanghai Medical College, Fudan University, Shanghai, China; ^5^ Department of Colorectal Surgery, Fudan University Shanghai Cancer Center, Shanghai, China

**Keywords:** colorectal cancer, gut microbiota, metabolite, metabolic pathway, amino acid metabolism

## Abstract

**Background:**

Tumor metabolomics of colorectal cancer (CRC) is significantly different from normal tissues, due to nutrient deprivation, metabolite accumulation, acidity and hypoxia. Besides, gut microbiota has been confirmed to affect the progression of CRC. Microbiota metabolites might participate in the metabolic reprogramming of CRC cells and further regulate tumor microenvironment.

**Method:**

10 CRC-related strains are cultured in vitro (10 replicates per bacterium), including Enterotoxic Escherichia coli (ETEC), Peptostreptococcus anaerobius (Pa), Fusobacterium necroporum (Fne), Fusobacterium nucleatum (Fn), Lactobacillus plantarum (Lp), Lactobacillus acidophilus (La), Lactobacillus casei (Lc), Lactobacillus rhamnosus gg (LGG), Bifidobacterium bifidum (Bbi), Bifidobacterium Breve (Bbr). Bacterial culture supernatant is subjected to gas chromatography-mass spectrometry.

**Result:**

The 10 CRC-related strains have characteristic metabolite profiles, mainly referring to specific saccharides, amino acids, bile acids, polyamines and bioactive compounds. Saccharides and organic acids increase significantly in *Lactobacillus* (Lp, LA, Lc and LGG) compared with culture medium and other strains, such as galactinol, 1-ketose, beta-gentiobiose, glutaric acid, 3-phenyllactic acid, indlol-3-lactate. Chlorogenic acid, a beneficial polyphenol, increases significantly in Bbr. The abundance of amino acids and their derivatives changes significantly in *Bifidobacterium* (Bbi and Bbr), such as 2-hydroxy-2-methylbutanoic acid, N-acetyl-5-hydroxytryptamine and glutamate. Bile acids (lithocholic acid and cholic acid), polyamine (spermine), amino acids and derivatives (N-acetylaspartate, glutamate) increased significantly in the CRC-related pathogens (ETEC, Pa, Fn and Fne). Correspondingly, metabolic pathways are significantly affected, mainly including amino acid metabolism and nucleotide metabolism.

**Conclusion:**

The 10 CRC-related strains possess significantly different metabolites and metabolic pathways. Specific metabolites and corresponding metabolic pathways might explain microbial CRC-promoting or -suppressing mechanisms.

## 1 Introduction

Since *Helicobactor pylori* was recognized as a gastrointestinal carcinogen, researchers have sought to elucidate the complicated relationship between microbiota and gastrointestinal diseases, particularly colorectal cancer (CRC). CRC ranks as the third most malignant tumor, causing estimated 1.8 million new cases and 881, 000 deaths annually according to epidemiologic findings (1). The intestine harbors a vast array of microorganisms and microbial dysbiosis participates in CRC pathogenesis. Consequently, interventions targeting gut microbiota-such as fecal microbiota transplantation, probiotics, prebiotics and specific diets- hold promising for improving the prognosis of CRC (2–6).

Specific microorganisms directly regulate CRC pathophysiology through physical interaction. For example, *Fusobacterium nucleatum*, a gram-negative anaerobic bacillus, promotes CRC development via adhesin FadA, Fap2 and RadD (7, 8). Similarly, *Peptostreptococcus anaerobius* drives CRC progression via the interaction between its surface protein (putative cell wall binding repeat 2) and α2/β1 integrin which is overexpressed in CRC cells (9). Simultaneously, gut microbiota, possessing potent metabolic capabilities, generates a significant quantity of metabolites. These metabolites are small molecules derived from bacterial fermentation of dietary substrates, modification of host secretory products, or direct microbial synthesis (10).

For example, short chain fatty acids (SCFAs) are produced by bacterial fermentation of dietary fiber (e.g., acetate, propionate and butyrate) (11). SCFAs protect epithelial barrier, regulate mucosal and systemic immunity (12), and directly suppress CRC cell survival, metastasis, angiogenesis through multiple signaling pathways (13).

Secondary bile acids are another crucial metabolites from gut microbiota, comprising deoxycholic acid (DCA), lithocholic acid (LCA) and ursodeoxycholic acid (UDCA). DCA and LCA damage the epithelial cells, induce abnormal inflammation, and promote CRC progression (14, 15), while the role of UDCA remains controversial (16, 17). Besides, polyamines mainly produced by *Bacteroides* and *Paraprevotella* exhibit different effect on CRC (e.g., putrescine, spermidine, spermine, agmatine). Commensal microbiota-derived agmatine promotes intestinal inflammation and tumorigenesis (18), whereas spermidine alleviates inflammatory bowel disease (IBD) severity and suppress CRC growth (19). Exploring microbial metabolic profile of microorganisms might assist us in recognizing and identifying more CRC-related metabolites.

We reviewed literature on CRC and microbiota, and carefully selected 10 CRC-related bacteria: 6 probiotics (*Lactobacillus plantarum* (Lp) (20, 21), *Lactobacillus acidophilus* (La) (22), *Lactobacillus casei* (Lc) (23), *Lactobacillus rhamnosus gg* (LGG) (24), *Bifidobacterium bifidum* (Bbi) (25), *Bifidobacterium Breve* (Bbr) (26)) and 4 pathogenic bacteria (*Enterotoxic Escherichia coli* (ETEC) (27), *Peptostreptococcus anaerobius* (Pa) (9), *Fusobacterium necroporum* (Fne) (28), *Fusobacterium nucleatum* (Fn) (29)). Altered abundance of these bacteria is also observed in other intestinal disorders, such as IBD and colorectal adenoma. Supplementing with these probiotics (Lp (30), La (31), Lc (32), LGG (33), Bbi (34) and Bbr (35)) reduces IBD severity. Furthermore, Fn is enriched in human colonic adenomas compared to adjacent tissue and in stool samples from adenoma/carcinoma patients versus healthy subjects (36, 37). Cui et al. also reported the reduced Bifidobacterium and increased Escherichia coli in patients with colorectal polyps compared to healthy individuals (38). These findings underscore the intimate connection between these 10 bacterial species and intestinal health. Investigating microbial metabolites hold promise for unveiling the mechanisms by which they regulate intestinal diseases. In this study, these bacteria were cultured *in vitro* (10 replicates per species) and then subjected to gas chromatography-mass spectrometry to identify characteristic metabolites.

## 2 Materials and methods

### 2.1 Bacterial strains and culture conditions

The 10 CRC-related strains included Lp (ATCC8014), La (ASI2686), Lc (ATCC334), LGG (BNCC134266), Bbi (ATCC29521), Bbr (ATCC15700), ETEC (BNCC195617), Pa (ATCC27337), Fne (ATCC51357), Fn (ATCC 25586). These strains used in this study were obtained from Tongji University Institute of Intestinal Diseases. Ethics committee of shanghai tenth people’s hospital affiliated to Tongji University did not require the study to be reviewed or approved by an ethics committee because no human sample and animal experiments were enrolled in this study. The same number of bacteria (1*10^6 CFU) were added to an equal volume of culture medium and incubated for 48 hours. Due to varying growth rates of bacteria, the concentration of bacteria differed when we collected the supernatant, despite the initial number of bacteria and the incubation time being consistent. Lp, La, Lc, LGG, Bbi and Bbr were cultured in de Man-Rogosa-Sharpe broth at 37 °C for 48h under anaerobic conditions(80% N2, 10% H2, 10% CO2). ETEC was cultured in Luria-Bertani broth at 37 °C for 48h under aerobic conditions. Pa, Fn and Fne were maintained in Wilkins-Chalgren anaerobe broth at 37 °C for 48h under anaerobic conditions. Each bacteria was culture 10 times. The supernatant of these bacteria and uninoculated media were subjected to gas chromatography-mass spectrometry (GC-MS).

### 2.2 Sample preparation

20 μL of 2-chloro-l-phenylalanine (0.3 mg/mL) dissolves in methanol as internal standard and 200 μL of sample is added to an Eppendorf tube of 1.5 mL, then dried in a freeze drier. 200 μL of methanol: acetonitrile (2: 1 = v: v) is added to each sample, dispersing sample by pipette. All of the mixtures of each sample are extracted by ultrasonication for 5 min in ice water bath. The samples are centrifuged at 12000 rpm for 10 min at 4°C. QC sample is prepared by mixing aliquots of the all samples to be a pooled sample. An aliquot of the 100 μL supernatant was transferred to a glass sampling vial for vacuum dry at room temperature. Subsequently, 80 μL of 15 mg/mL methoxylamine hydrochloride in pyridine is added. The resultant mixture is vortexed vigorously for 2 min and incubated at 37 °C for 90 min. 80 μL of BSTFA (with 1% TMCS) and 20 μL n-hexane are added into the mixture, which is vortexed vigorously for 2 min and then derivatized at 70 °C for 60 min. The samples are placed at ambient temperature for 30 min before GC MS analysis.

### 2.3 Gas chromatography-mass spectrometry

The derivative samples are analyzed on an Agilent 7890B gas chromatography system coupled to an Agilent 5977A MSD system (Agilent Technologies Inc., CA, USA). A DB-5MS fused silica capillary column (30 m × 0.25 mm × 0.25 μm, Agilent J & W Scientific, Folsom, CA, USA) is utilized to separate the derivatives. Helium (> 99.999%) is used as the carrier gas at a constant flow rate of 1 mL/min through the column. The injector temperature is maintained at 260 °C. Injection volume is 1 μL by splitless mode. The initial oven temperature is 60 °C, ramped to 125 °C at a rate of 8 °C/min, to 210 °C at a rate of 5 °C/min, to 270 °C at a rate of 10 °C/min, to 305 °C at a rate of 20 °C/min, and finally held at 305 °C for 2 min. The temperature of MS quadrupole and ion source (electron impact) is set to 150 and 230 °C, respectively. The collision energy is 70 eV. Mass spectrometric data is acquired in a full scan mode (m/z 50-500). The QCs are injected at regular intervals throughout the analytical run to provide a set of data from which repeatability could be assessed.

### 2.4 Cell culture

Human colorectal cancer cell HCT116 is obtained from Cell Culture Bank of the Chinese Academy of Sciences (Shanghai, China). HCT116 cells are cultured in DMEM medium (Gibco Life Technologies, USA) added with 10% FBS (Gibco Life Technologies, USA).

### 2.5 CCK-8 assay and colony formation assay

Cells are digested into a single-cell state and seeded into 96-well plates (3000 cells/well). A 10% Cell Counting Kit-8 reagent (Yeasen, China) is added and CRC cells are incubated for 1 h to obtain OD value of 450nm. Cells are seeded into 6-well plates (500 cells/well) for 10 days. Then cells are washed with PBS, fixed by formaldehyde, and stained with 0.1% crystal violet.

### 2.6 Migration and invasion assays

For migration assays, 20,000 cells/200µL FBS-free medium are added into transwell chambers (Yeasen, China). Each well contained 600 µL medium with 10% FBS in 24-well plates (Yeasen, China). After 24h, cells were fixed with formaldehyde, stained with

0.1% crystal violet, and imaged. The invasion assay follows the same procedure, except Matrigel (Yeasen, China) was applied to the chamber surface before cell seeding.

### 2.7 Apoptosis assay

CRC cells are seeded into 6-well plate (300,000 cells/well) and cultured for 24h. Then metabolites are added in wells and cells are cultured for 24h. After washing twice with PBS, cells are processed using a flow apoptosis kit (Vazyme, China) and analyzed by flow cytometry (BD Biosciences, USA).

### 2.8 Statistical analysis

The raw data (.D format) is converted to.CDF format utilizing ChemStation (version E.02.02.1431, Agilent, USA) software and then imported into the ChromaTOF software (version 4.34, LECO, St Joseph, MI) for data processing. Metabolites are annotated utilizing Fiehn or NIST database. After alignment with Statistic Compare component, the ‘raw data array’ (.cvs) is obtained from raw data with 3-dimension data sets including sample information, peak names (or retention time and m/z) and peak intensities.

In the ‘data array’, all internal standards and any known pseudo positive peaks (caused by background noise, column bleed or BSTFA derivatization procedure) are removed. The data is normalized to the total peak area of each sample, and multiplied by 10000, and the peaks from the same metabolite are combined. Principle component analysis (PCA) and (orthogonal) partial least squares discriminant analysis ((O)PLS-DA) are performed to visualize the metabolic difference among experimental groups. Variable importance in the projection (VIP) ranks the overall contribution of each variable to the OPLS-DA model, and those variables with VIP > 1 are considered relevant for group discrimination. To visually demonstrate the trend of change, we utilized fold change to show the changed metabolites between the supernatant of bacteria and culture medium. There is a statistically significant difference between the two groups according to VIP>1and *p* value of t test <0.05.

## 3 Result

### 3.1 Distinct metabolite profiles revealed by PCA, PLS-DA, and OPLS-DA

**Figure 1:**
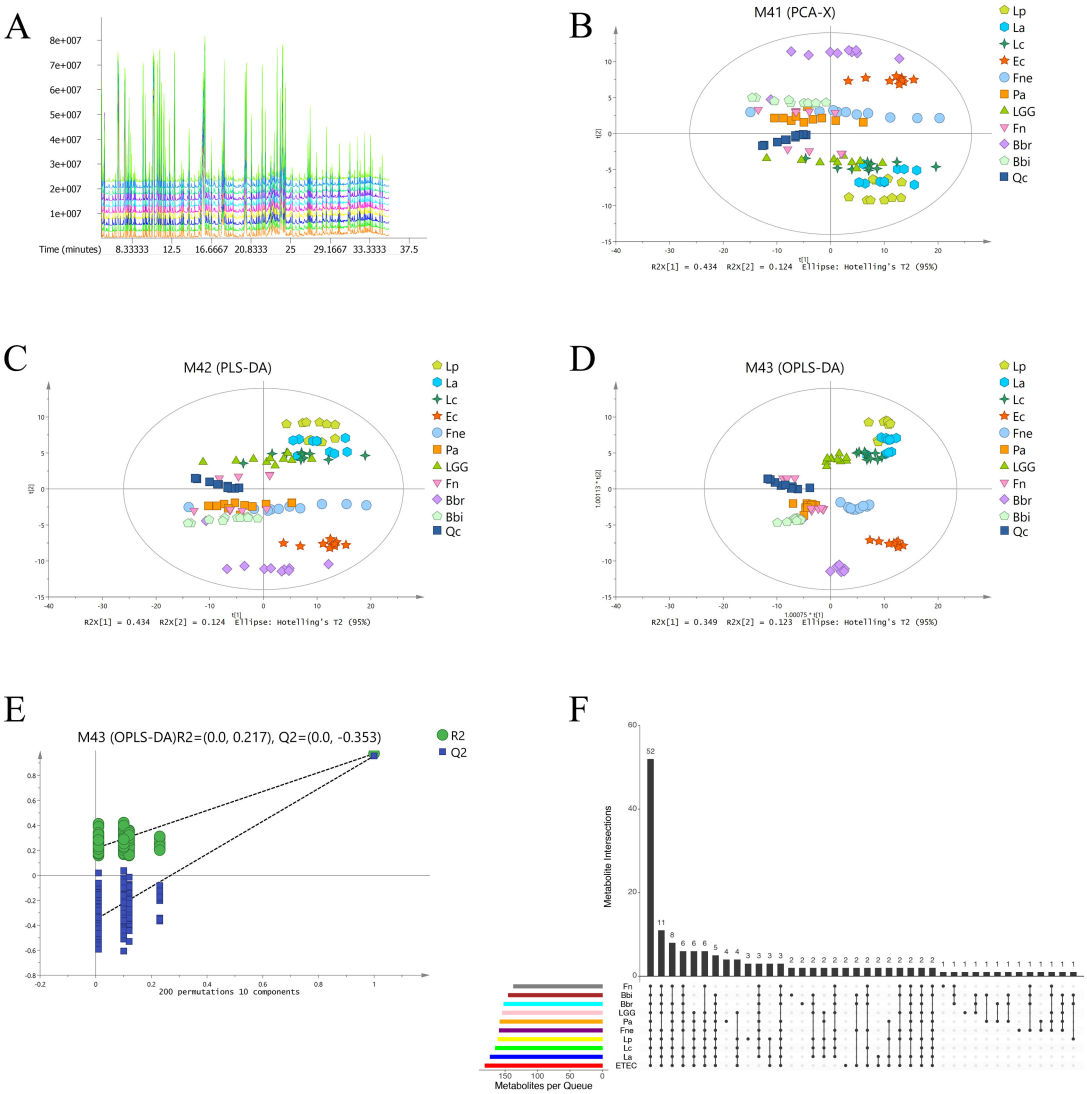
The 10 CRC-related bacteria had different metabolite profiles. **(A)** Total ion chromatogram macroscopically reflected the metabolite separation in GC-MS. **(B)** Principal component analysis of microbial metabolites. **(C)** Partial least squares discriminant analysis of microbial metabolites. **(D)** Orthogonal partial least squares discriminant analysis of microbial metabolites. **(E)** The response permutation testing of orthogonal partial least squares discriminant analysis. **(F)** Upset plot of metabolites identified in the different bacteria.

The total ion chromatogram (TIC) provided an overview of metabolite separation in the GC-MS analysis (**Figure 1**). Principal component analysis (PCA), partial least squares discriminant analysis (PLS-DA), and orthogonal partial least squares discriminant analysis (OPLS-DA) collectively demonstrated distinct metabolite profiles for the 10 bacterial species, as evidenced by their separation into different regions of the score plots (**Figure 1B**). Notably, the *Lactobacillus* species (La, Lp, Lc, and LGG) clustered predominantly in the delta quadrant while Bifidobacterium species (Bbi and Bbr) were located mainly in the alpha and beta quadrants. This distinct spatial distribution, consistent across PLS-DA and OPLS-DA models, suggested an association between metabolite profiles and phylogenetic relationships. The close clustering of quality control (QC) samples in the PCA, PLS-DA, and OPLS-DA score plots confirmed the stability and repeatability of the experimental data. The response permutation testing showed that OPLS-DA models of the 10 bacteria were stable and not over-fitting (**Figure 1**).

### 3.2 The differential metabolites of the 10 CRC-related bacteria

The differential metabolites were mainly composed of sugar and derivatives, amino acids, bile acids, polyamines, bioactive compounds. According to variable important in projection (VIP)>1, *p*<0.05 compared with culture medium (CM), the significantly different metabolites are screened out, including 161 metabolites of Lp, 173 metabolites of La, 165 metabolites of Lc, 154 metabolites of LGG, 145 metabolites of Bbi, 152 metabolites of Bbr, 181 metabolites of ETEC, 158 metabolites of Pa, 159 metabolites of Fne and 137 metabolites of Fn (**Figure 1**, **Supplementary Table 1**).

#### 3.2.1 Lp, La, Lc and LGG

**Figure 2:**
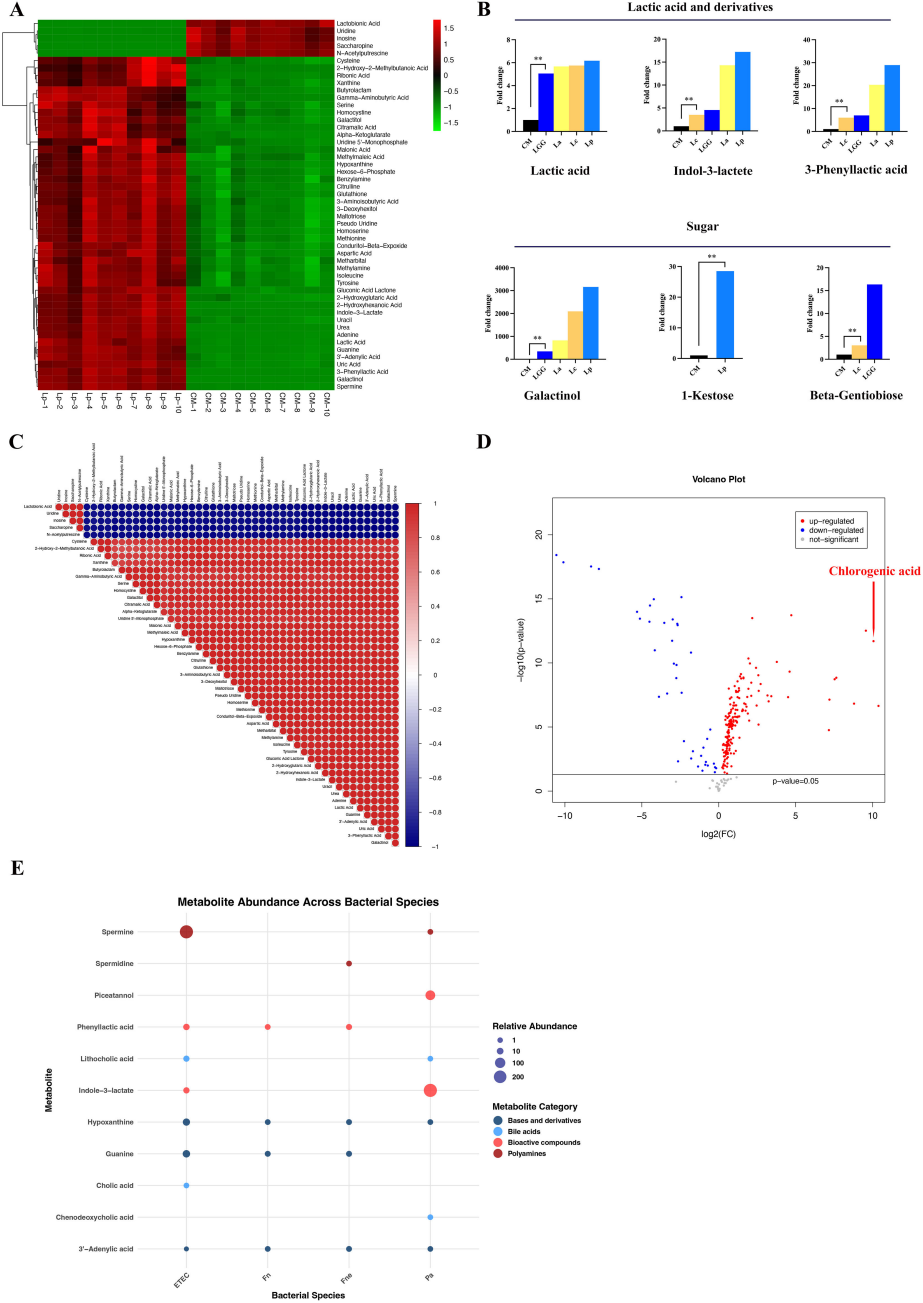
The characteristic metabolites of the 10 CRC-related bacteria. **(A)** The heat map of top 50 metabolites from Lp. The heat maps of La, Lc and LGG were displayed in **Supplementary Figure 1**. **(B)** The histogram showed the increased content of lactate acid and derivatives as well as sugar in *Lactobacillus*, especially in Lp and La. **(C)** The correlation analysis of metabolites from Lp. The correlation analysis of La, Lc and LGG was shown in **Supplementary Figure 2**. **(D)** The volcano plot indicated that chlorogenic acid was increased significantly in the supernatant of Bbr compared to culture medium. **(E)** The bubble plot displayed the increased content of bile acids, ployamines, bioactive compounds, bases and their derivatives in CRC-related pathogens (ETEC, Pa, Fne, Fn). **p<0.01.

The content of organic acids increased significantly in the supernatant of *Lactobacillus*, especially Lp and La (**Figure 2**, **Supplementary Figure 1**). The content of lactic acid increased in Lp, La, Lc and LGG (FC:6.17, 5.67, 5.75, 5.06 respectively, *p*<0.01). Lactic acid derivatives were enriched significantly in Lp and La, such as 3-phenyllactic acid (FC:28.94, 20.35 respectively, *p*<0.01), indole-3-lactate (FC:17.23, 14.29 respectively, *p*<0.01) (**Figure 2**). Correlation analysis demonstrated a positive correlation between lactic acid and its derivatives, indicating that *Lactobacillus* possessed the capability to further metabolize lactic acid (**Figure 2**, **Supplementary Figure 2**). Besides, citramalic acid increased significantly in Lp and LGG (FC:10.94, 10.31 respectively, *p*<0.01). Lp also had increased gamma-aminobutyric acid (FC:23.64, *p*<0.01) and n-acetylglycine (FC:86.72, *p*<0.01) while Lc possessed increased glutaric acid (FC:24.0, *p*<0.01).

The content of saccharides and derivatives changed significantly in La, Lc, Lp and LGG (**Figure 2**). A hundred-fold increase of galactinol was found in Lp, Lc, La and LGG compared with that in CM (fold change (FC): 3160.48, 2094.59, 823.78, 344.82 respectively, *p*<0.01) while the content of galactinol did not change in the other strains. 1-Kestose, a trisaccharide, increased significantly in Lp (FC: 28.52, *p*<0.01) while beta-gentiobiose, a glycosylglucose, increased significantly in LGG compared to CM (FC:16.36, *p*<0.01). Besides, Lc and LGG reduced the content of maltotriitol (FC: 0.088, 0.065 respectively, *p*<0.01). Collectively, saccharides, organic acids, and their derivatives are enriched in the supernatant of specific *Lactobacillus* spp. compared with CM and other bacteria.

#### 3.2.2 Bbi and Bbr

We observed a dramatic increase of chlorogenic acid (CGA) content in Bbr supernatant compared with CM (FC:1339.25, *p*<0.01) (**Figure 2**, **Supplementary Table 2**).

The content of amino acids decreased significantly in the supernatant of Bbi and Bbr compared to CM, including tyrosine (FC: 0.003, 0.003 respectively, *p*<0.01), norleucine (FC: 0.16, 0.15 respectively, *p*<0.01), citrulline (FC: 0.15, 0.13 respectively, *p*<0.01) and trans-4-hydroxy-L-proline (FC: 0.11, 0.04 respectively, *p*<0.01), indicating that Bbi and Bbr possessed the function of metabolizing amino acid. Correspondingly, the abundance of amino acids derivatives increased in the culture supernatant of Bbi and Bbr compared to CM, such as 2-hydroxy-2-methylbutanoic acid (FC:13.54, 24.27 respectively, *p*<0.01) and n-acetyl-5-hydroxytryptamine (FC:986.23, 1065.18 respectively, *p*<0.01).

#### 3.2.3 ETEC, Pa, Fne and Fn

Obviously, n-acetylaspartate, one cancer-related biomarker, increased significantly in ETEC supernatant (FC: 9798.52, *p*<0.01) compared with CM. Besides, n-acetyl-5-hydroxytryptamine (FC: 1772.45, 1632.89 respectively, *p*<0.01) and glutamate (FC: 1335.54, 1110.34 respectively, *p*<0.01) increased significantly in ETEC and Fne supernatants versus CM.

Significant increases in primary and secondary bile acids were observed in ETEC and Pa supernatants. ETEC supernatant exhibited elevated levels of cholic acid (FC: 1.88, *p*<0.01) and lithocholic acid (FC: 5.1, *p*<0.01). In Pa supernatant, increases were observed for lithocholic acid ((FC: 1.69, *p*<0.01)) and chenodeoxycholic acid (FC: 1.83, *p*<0.01) (**Figure 2**).

Polyamines, which were consist of putrescine, spermidine and spermine, took part in cell proliferation and differentiation (25, 26). We found that spermine increased significantly in ETEC supernatant (FC: 232.69, *p*<0.01) while spermidine increased in Fne supernatant (FC: 1.92, *p*<0.01) compared to CM.

Partial bioactive compounds, produced by fermentation of aromatic acids, were uncovered to change significantly in the selected CRC-related strains, including piceatannol, indole-3-lactate and phenyllactic acid. Piceatannol, a natural stilbene, increased significantly in Pa (FC: 78.96) compared with CM. We also find the increased indole-3-lactate in Lp (FC: 17.23, *p*<0.01), La (FC: 14.29, *p*<0.01), Lc (FC:3.51, *p*<0.01) and LGG (FC: 4.55, *p*<0.01). However, the more content of indole-3-lactate was found in the pathogen Pa compared with CM (FC:232.03, *p*<0.01). Besides, ETEC, Fne and Fn had increased 3-phenyllactic acid in contrast to CM(FC: 7.21, 3.92, 2.36, respectively), which was more in Lp (FC: 28.94, *p*<0.01), consistent with Zhou’s findings (27).

In ETEC, the content of bases and their derivatives changed significantly, such as guanine (FC: 21.12, *p*<0.01), hypoxanthine (FC: 17.66, *p*<0.01) deriving from an adenine, uridine (FC: 0.01, *p*<0.01) and 3’-adenylic acid (FC: 0.09, *p*<0.01) deriving from an adenosine. In addition, 2-hydroxy-2-methylbutanoic acid, a branched-chain fatty acid, increased in the pathogens, especially in Pa (FC: 68.19, *p*<0.01). 1-Monostearin increased significantly in Fn and Fne (FC: 28.24, 40.0 respectively, *p*<0.01).

**Figure 3:**
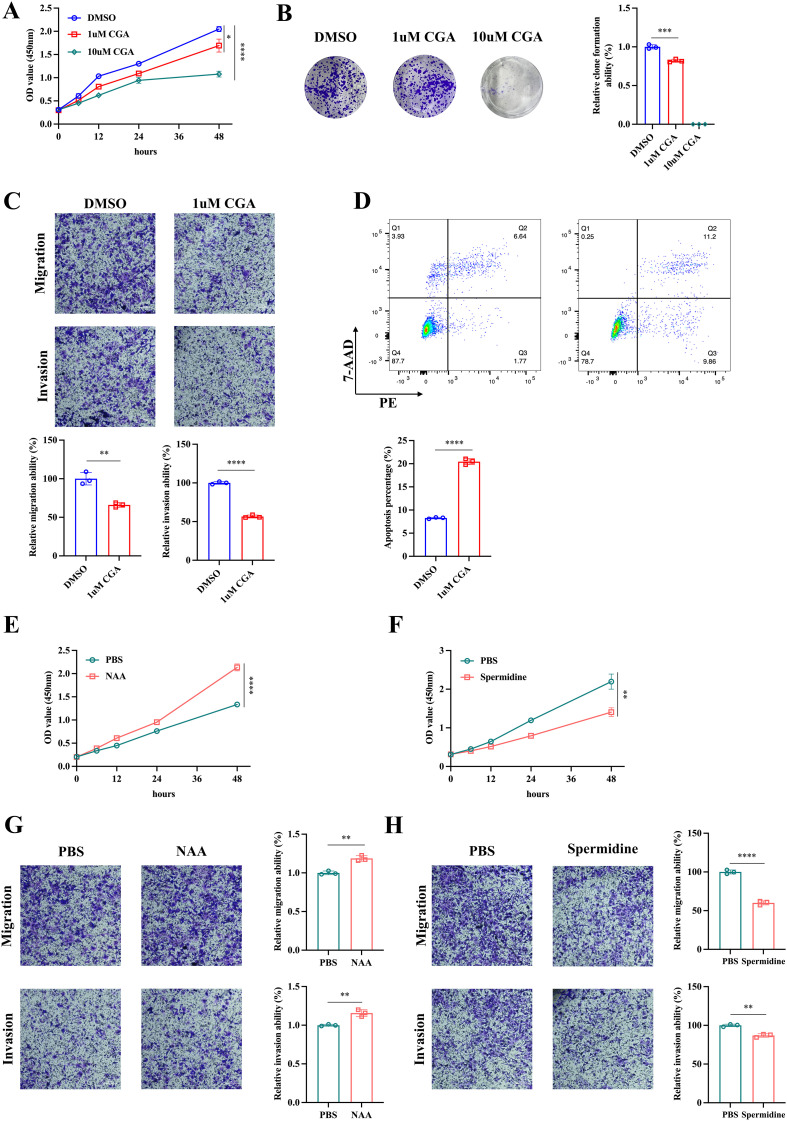
The effect of metabolites on CRC cells. The cck-8 assay **(A)** and colon formation assay **(B)** were conducted to assess the effect of chlorogenic acid (CGA) on CRC cell HCT116 proliferation. The transwell assay **(C)** and flow cytometry **(D)** were used to analyze HCT116 cell invasion, migration and apoptosis. The cck-8 assay **(E, F)** and transwell assay **(G, H)** were utilized to investigate the effect of n-acetylaspartate (NAA) and spermidine on HCT116 proliferation, invasion and migration. *p<0.05,**p<0.01,***p<0.001,****p<0.0001.

**Figure 4:**
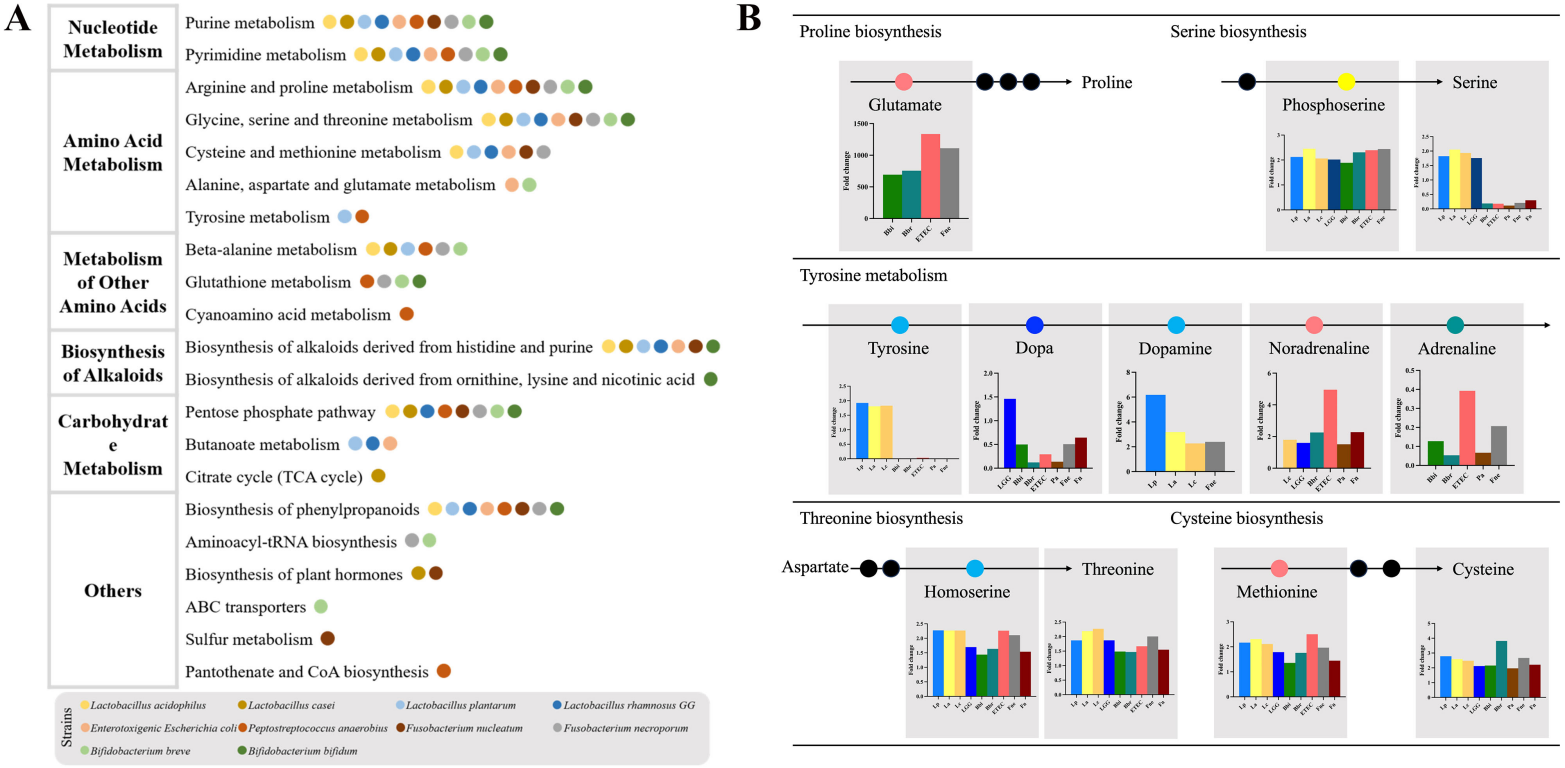
The discriminatory metabolic pathways of the CRC-related bacteria. **(A)** The pathway enrichment analysis. The histograms of top 10 metabolic pathways were displayed in **Supplementary Figure 3**. **(B)** The affected amino acid metabolic pathway.

Subsequently, we conducted *in vitro* experiments to investigate the effect of these metabolites on CRC cells. The CCK-8 assay and colony formation assay indicated that CGA significant suppressed CRC cell HCT116 proliferation (**Figures 3**, **4B**). The transwell assay and flow cytometry indicated that CGA also restrained HCT116 migration, invasion and promoted cell apoptosis (**Figures 3**, **4D**). Besides, NAA promoted CRC cell proliferation, migration and invasion whereas spermidine suppressed CRC cell proliferation, migration and invasion (**Figures 3E**).

### 3.3 The discriminatory metabolic pathways

The metabolic pathway enrichment analysis showed the top 10 metabolic pathways, which were mainly composed of nucleotide metabolism, amino acid metabolism, metabolism of other amino acids, biosynthesis of alkaloids and carbohydrate metabolism (**Figure 4**, **Supplementary Figure 3**).

The discriminatory amino acid metabolism pathways comprised the arginine and proline metabolism, the glycine, serine and threonine metabolism, the cysteine and methionine metabolism, the alanine, aspartate and glutamate metabolism as well as the tyrosine metabolism (**Figure 4**). The metabolism of other amino acids included the beta-alanine metabolism, the glutathione metabolism and the cyanoamino acid metabolism. Bbi, Bbr, ETEC and Fne promoted an increase in glutamate, the substrate for proline synthesis. In the process of serine synthesis, these bacteria were all capable of increasing phosphoserine, except for Pa and Fn. However, these CRC-promoting bacteria (ETEC, Pa, Fn and Fne) consumed serine whereas *Lactobacillus* (Lp, La, Lc and LGG) increased the content of serine. In the process of tyrosine metabolism, *Lactobacillus* increased the content of tyrosine, dopa and dopamine while adrenaline was reduced by *Bifidobacterium* and CRC-promoting bacteria. Besides, these CRC-related bacteria could affect the threonine biosynthesis and cysteine biosynthesis.

The differential nucleotide metabolism pathway included purine metabolism and pyrimidine metabolism, which were further composed of *de novo* biosynthetic pathway, salvage pathway and degradation pathway. Partial metabolites of these CRC-related bacteria were vital substrates of purine and pyrimidine metabolism.

## 4 Discussion

Advances in sequencing technology and bioinformatics have revealed microbial dysbiosis in numerous gastrointestinal and systemic diseases, such as colorectal cancer, inflammatory bowel disease, Alzheimer’s disease and et al. (10, 39, 40). However, the mechanism by which microorganisms contribute to disease progression remain incompletely understood. We investigated the underlying mechanism of bacteria regulating the process of CRC from the perspective of microbial metabolites in this article. Our analysis identified characteristic metabolites and significantly altered metabolic pathways in supernatants from both beneficial bacteria and detrimental pathogens. We observed a significant increase in saccharides in *Lactobacillus* supernatant, such as 1-kestose, galactinol, beta-gentiobiose. 1-Kestose, the smallest fructooligosaccharide, was enriched in Lp supernatant. Previous studies indicated that 1-kesose could improve gut microbiota. For example, 1-Kestose could activate butyrate-produced bacteria, such as *Clostridium cluster IV*, *Clostridium cluster XIVa* and *Bacteroidetes* and promote the production of SCFAs (41, 42). Akihito also found that the supplement of 1-Kestose increased the abundance of *Bifidobacterium* and suppressed *Escherichia coli* in the fecal batch culture model of human adult *in vitro* (43). Besides, administration of 1-Kestose was found to significantly increase the population of CD4^+^Foxp3^+^ cells in mesenteric lymph nodes (MLNs) (44), suggesting that 1-kestose might suppress inflammation-induced CRC by regulating intestinal immunity (**Supplementary Figure 4**). We also found a hundreds-fold or thousands-fold increment of galactinol in *Lactobacillus* (Lp, La, Lc and LGG). However, there is still lack of studies focused on the role of galactinol produced by *Lactobacillus* in CRC-related microenvironment.

The concentration of organic acids changed significantly in *Bifidobacterium*. Chlorogenic acid (CGA), a polyphenol comprised of caffeic acid and quinic acid, increased significantly in Bbr. CGA possesses the antioxidant, anti-inflammation, anti-diabetic and anti-obesity properties (45, 46). Regarding to CRC, CGA could suppress the viability and migration of cancer cells by promoting ROS production, reducing miR-31 oncogene, arresting the cell cycle, suppressing NF-κB pathways and increasing cytotoxicity (47–49). According to Jiang’s research, CGA effectively suppressed hepatoma by inducing cancer cell differentiation (50). Besides, studies revealed that CGA has antimicrobial properties, such as *Escherichia coli*, *Helicobacter pylori*, *Staphylococcus epidermidis* (51, 52). Briefly, Bbr might suppress the pathogenesis of CRC by producing chlorogenic acid and then regulating multiple pathways, but noteworthily, this suppression might be concentration-dependent.

We screened out the significantly changed metabolites from the 4 pathogens (ETEC, Pa, Fn and Fne), such as amino acids and derivatives (N-acetylaspartate (NAA), glutamate), bile acids, polyamines (putrescine, spermidine, and spermine), bioactive compounds (piceatannol, indole-3-lactate and phenyllactic acid). NAA was significantly enriched in ETEC. Aspartate N-acetyltransferase (NAT8L), a NAA biosynthetic enzyme, was negatively associated with overall survival duration in CRC patients, as shown in the cancer genome atlas (TCGA) (53). Ovarian cancer cells could release NAA to sustain M2-like macrophage by enforcing the glutamine synthetase expression of macrophage (54). Ovarian cancer cell viability and proliferation are suppressed by silencing NAT8L and reversed by adding NAA (53). Besides, increased NAA was also identified in non-small cell lung cancer (55) and glioblastoma (56). Thereby, NAA produced by ETEC might affect the immune microenvironment and promote CRC progression. The high level of glutamate was found in ETEC and Fne. It has been confirmed that high-level glutamate promoted inflammation and induced obesity and diabetes, both of which were associated with early-onset colorectal cancer in animal models (57, 58).

Enriched bile acids were found in ETEC and Pa, including cholic acid, chenodeoxycholic acid (CDCA) and lithocholic acid (LCA). Primary bile acids that compose of cholic acid and chenodeoxycholic acid is mainly synthesized in liver from cholesterol. Cholic acid and CDCA are transformed into deoxycholic acid (DCA) and LCA respectively by gut microbiota. Hydrophobic bile acids (DCA and LCA) deteriorated CRC via multiple pathways, such as producing reactive oxygen species and reactive nitrogen substances, activating EGFR-MAPK pathway, regulating M3R and Wnt/beta-catenin signaling (59).

Polyamines, mainly composed of putrescine, spermidine and spermine, were enriched in the tumor microenvironment of CRC (60). We found that spermine increased in ETEC (FC: 232.69) while spermidine increased in Fne (FC: 1.92). However, spermine and spermidine had significantly different effect on tumor microenvironment. Spermidine promoted macrophage M1 polarization whereas spermine favored macrophage M2 polarization (61). Spermidine supplement improved antitumor immunity via combining with mitochondrial trifunctional protein (MTP), then increasing fatty acid oxidation and activating CD8^+^T cells (60). Besides, spermine could inhibit fatty acid oxidation caused by spermidine by competitively combining with MTP. Thereby, polyamines produced by ETEC and Pa might regulate the balance of spermine and spermidine in tumor microenvironment and impact the immunotherapy for CRC.

Interestingly, anti-cancer bioactive compounds were enriched in Pa, including piceatannol, indole-3-lactate, although Pa was considered as a detrimental pathogen for CRC. Piceatannol could arrest tumor cell at S stage and suppress M2-polarized tumor-related macrophage to inhibit CRC progression (62, 63). Besides, Piceatannol inhibited other malignant tumors by restraining PI3K/AKT/mTOR pathway and decreasing COX2 (64–66). Indole-3-lactate, another bioactive compound, was also found to suppress CRC effectively (67). So, Pa might have a double-edged effect on CRC and tumor treatment due to specific metabolites.

The affected metabolic pathways were observed in the different bacteria, such as nucleotide metabolism, amino acid metabolism, metabolism of other amino acids, biosynthesis of alkaloids and carbohydrate metabolism.

Nucleotide metabolism provided essential purine and pyrimidine nucleotide for DNA and RNA biosynthesis, and energy and cofactor for cell survival, which were all required in CRC cells. Both of purine and pyrimidine metabolism were composed of *de novo* biosynthetic pathway, salvage pathway and degradation. The content of important substrate of purine and pyrimidine metabolism pathways changed significantly among the different bacteria. For example, inosine increased significantly in Lc, LGG and Pa while adenosine increased significantly in Lp and Fn regarding purine salvage pathway.

Soares and his colleagues revealed that inosine potently enhanced the melanoma cell proliferation (68). The ratio of adenosine to inosine was also related to tumor growth, invasiveness and metastasis (69). Several amino acids (glutamine, aspartate, glycine) and one-carbon units were necessary for generation of inosine monophosphate (IMP) in *de novo* purine biosynthetic pathway. Glutamine, which increased significantly in Fne, was vital for cancer cells, such as sustaining proliferation signal and immortality, providing energy, assisting invasion and metastasis (70). Aspartate decreased significantly in Bbr and ETEC whereas it increased in other strains. Glycine increased in Fne, Pa, La, Lp and Bbr. Aspartate and glycine were necessary for specific cancer cells and the activation of aspartate and glycine biosynthetic pathway might promote oncogenesis (71–73).

CRC cells with a hallmark of rapid proliferation and huge nutritional consumption are usually within a nutrient-poor environment, especially lack of amino acids and need uptake of nutritional substrates from environment. Besides, normal intestinal epithelial cells also need supplement of exogenous nutrition. For example, glutamine, the content of which increased significantly in Fne, was required for the biosynthesis of glucosamine-6-phosphate and nonessential amino acids and for uptake of several essential amino acids (74). The significantly changed content of amino acids might be capable of regulating the amino acids metabolism pathways in CRC cells. However, the nutrient uptake from extracellular fluid was regulated by multiple approaches, such as growth factor signaling, genetic alteration and interaction between cells and extracellular matrix (75, 76). Therefore, further research is needed to ascertain whether the amino acids produced by microorganisms can influence CRC progression.

There are some deficiencies in this study. We have not yet verified the correlation between these CRC-related bacteria and metabolites in clinical samples. There is a lack of animal experiments to verify the effect of differential metabolites on tumor progression. Initially, we attempted to cultivate these 10 bacteria using a single culture medium (LB, brain heart infusion broth), but failed. We will continue to explore whether it is possible to combine these bacteria in a single culture medium. Besides, We tried to explore the correlation between these CRC-related bacteria and metabolites in clinical samples utilizing 3 cohorts of CRC patients and healthy individuals. In Cohort 1, fecal samples of 50 CRC patients and 50 healthy volunteers were subjected to 16S rRNA gene sequencing and untargeted metabolomics by GC-MS (77). In Cohort 2, serum metabolomics by LC-MS and metagenome sequencing of paired fecal samples were applied to identify gut microbiome-associated metabolites in 49 CRC patients and 31 healthy individuals (78). In Cohort 3, serum metabolomics were analyzed in 34 healthy individuals and 35 ones with CRC (79). However, due to the relatively low abundance of these CRC-related bacteria and metabolites, untargeted metabolomics could not identify these metabolites, such as CGA, NAA, 3-phenyllactic acid, spermidine, piceatannol and so on in Cohort 1 and Cohort 2. We found that the concentration of NAA, glutamate and spermidine had no significant difference between CRC patients and healthy ones in Cohort 3. In subsequent research, we will utilize targeted metabolomics to measure the levels of these metabolites in CRC patients’ feces and blood, and explore their correlation with microorganisms.

## 5 Conclusion

The 10 CRC-related strains have specific metabolites, primarily encompassing saccharides, organic acids, polyamines, bile acids, bioactive compounds. Specific metabolites and influenced metabolite pathways showed significant associations with CRC status.

## Data Availability

The original contributions presented in the study are included in the article/**Supplementary Material**. Further inquiries can be directed to the corresponding authors.

## Supplementary material

Supplementary Figure 1 The heat maps of The heat maps of top 50 metabolites from La, Lc and LGG compared to CM.

Supplementary Figure 2 The correlation analysis of metabolites from La, Lc and LGG.

Supplementary Figure 3 The pathway enrichment analysis revealed the top 10 metabolic pathways.

Supplementary Figure 4 Characteristic metabolites and possible CRC-regulating mechanism of the 10 bacteria. ↑: promoting, ⊥:suppressing. Full line means these metabolites or pathways are confirmed by our study or other researches. Dotted line means the regulated pathways are found in other diseases and have not been confirmed in CRC. CRC, colorectal cancer; SCFAs, short chain fatty acids; MLNs, mesenteric lymph nodes; ROS, reactive oxygen species; BCs, bile acids; CDCA, chenodeoxycholic acid; LCA, lithocholic acid; DCA, deoxycholic acid.

Supplementary Table 1 The different metabolites of the 10 bacteria.

Supplementary Table 2 The different metabolites of Bbr compared to culture medium.
