# Supplementary figures and images for: Characteristic metabolite profile of 10 colorectal cancer-related bacteria
Source: Front Oncol. 2025 Jul 14;15:1604876. doi: 10.3389/fonc.2025.1604876 (PMC12301220; doi:10.3389/fonc.2025.1604876)

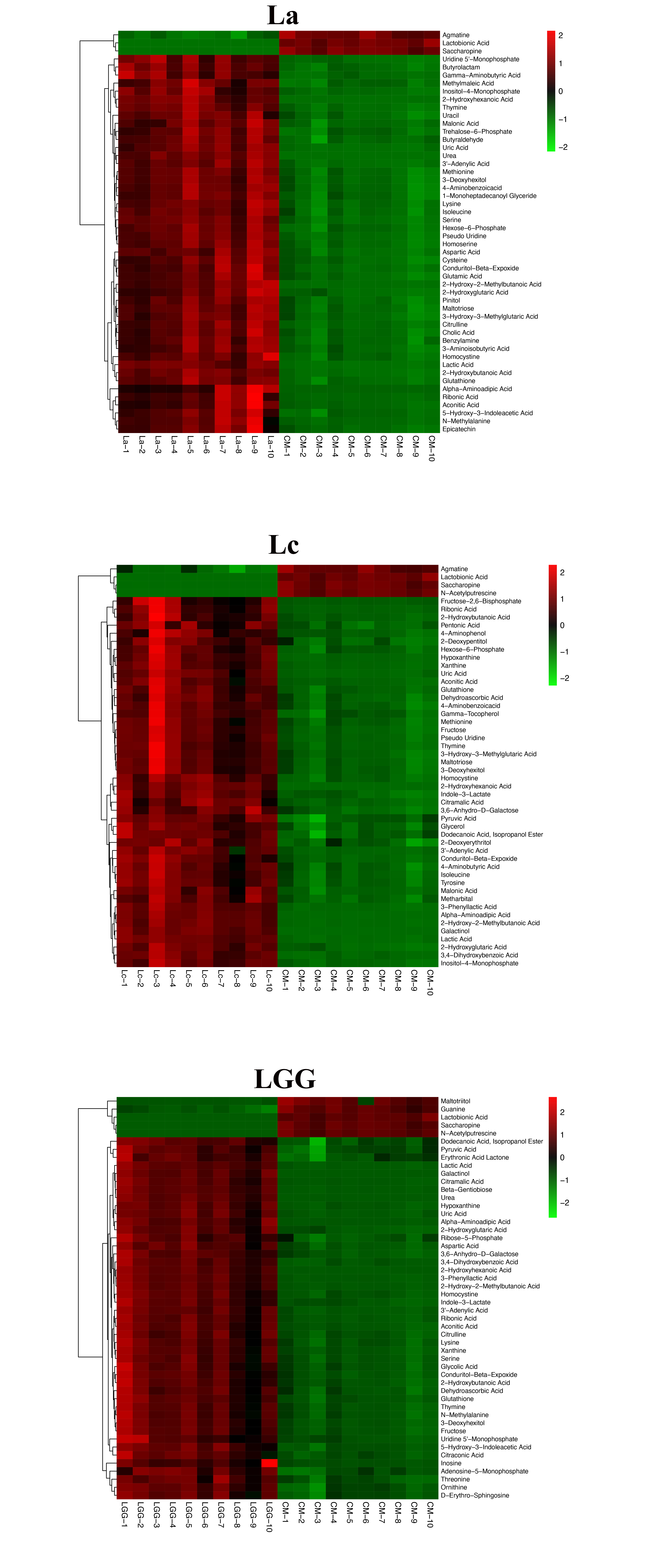

Supplement: Supplementary Figure 1 — The heat maps of The heat maps of top 50 metabolites from La, Lc and LGG compared to CM. [file Image1.jpeg]

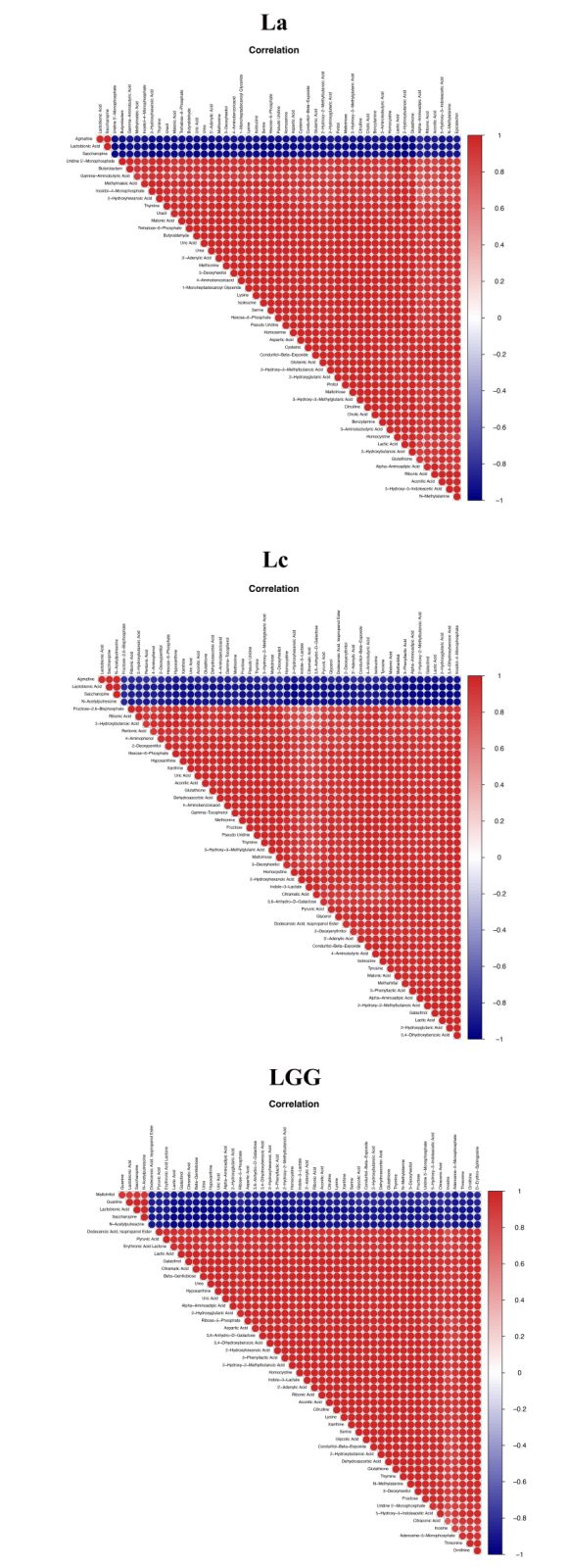

Supplement: Supplementary Figure 2 — The correlation analysis of metabolites from La, Lc and LGG. [file Image2.jpeg]

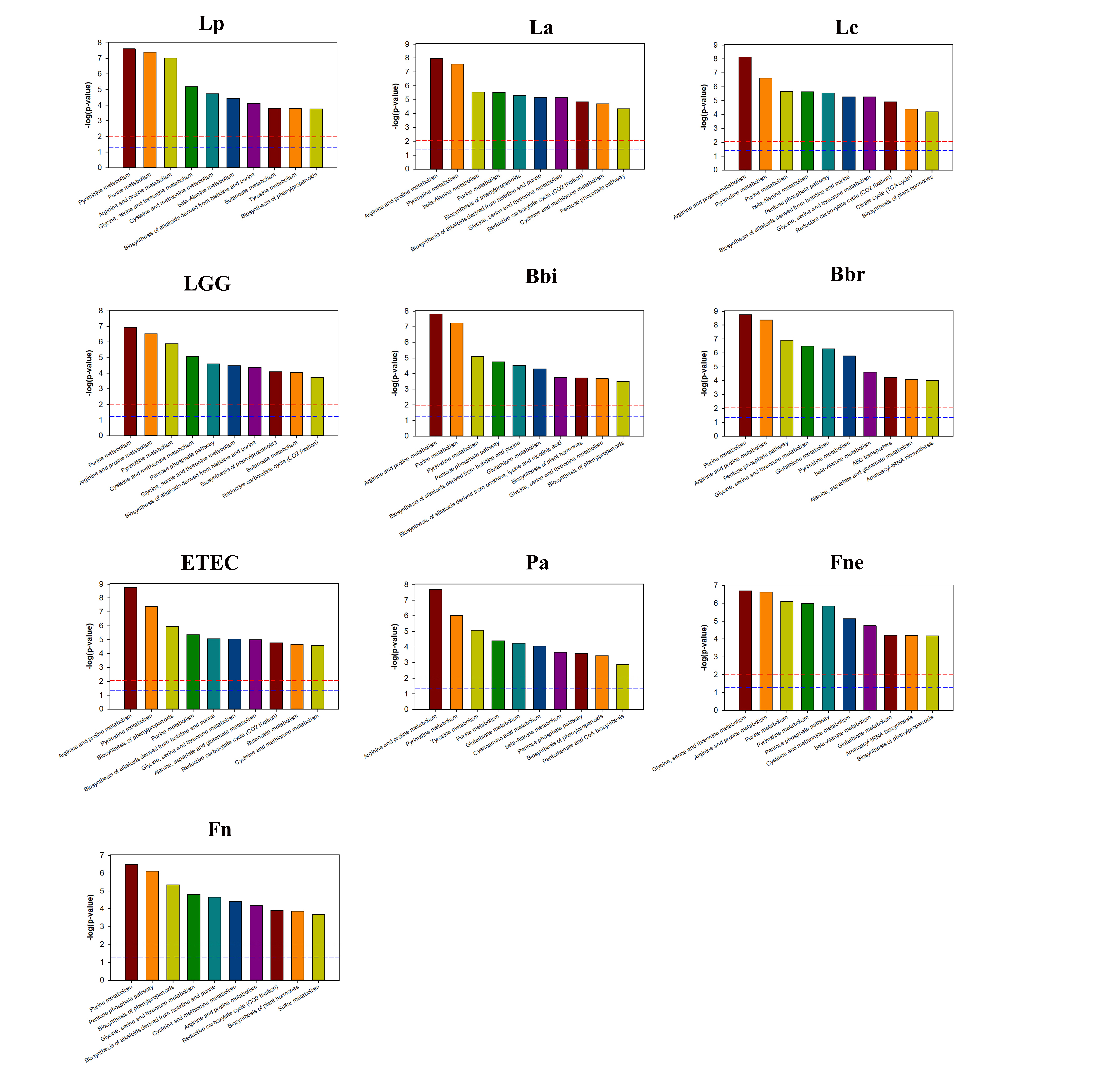

Supplement: Supplementary Figure 3 — The pathway enrichment analysis revealed the top 10 metabolic pathways. [file Image3.jpeg]

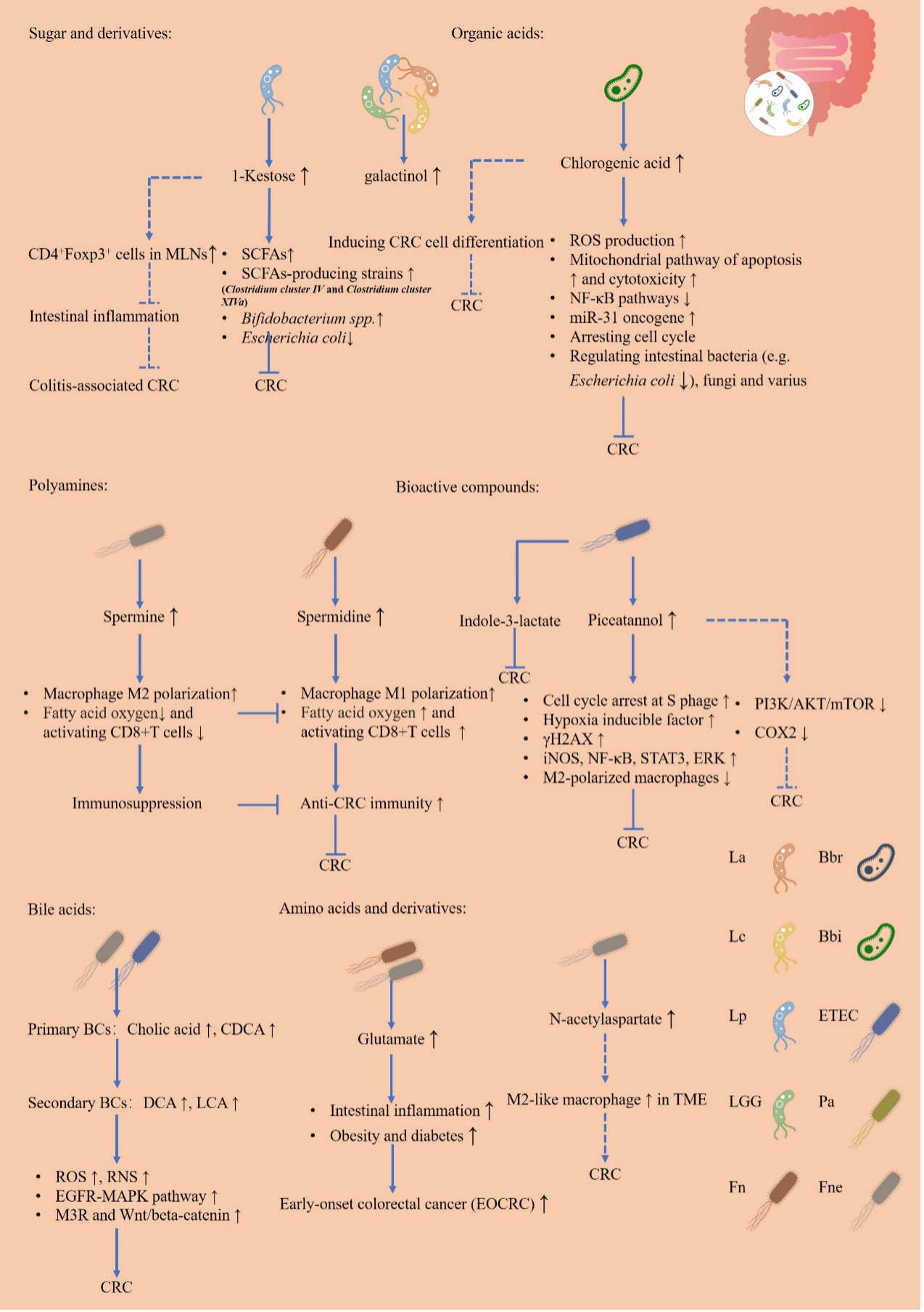

Supplement: Supplementary Figure 4 — Characteristic metabolites and possible CRC-regulating mechanism of the 10 bacteria. ↑: promoting, ⊥:suppressing. Full line means these metabolites or pathways are confirmed by our study or other researches. Dotted line means the regulated pathways are found in other diseases and have not been confirmed in CRC. CRC, colorectal cancer; SCFAs, short chain fatty acids; MLNs, mesenteric lymph nodes; ROS, reactive oxygen species; BCs, bile acids; CDCA, chenodeoxycholic acid; LCA, lithocholic acid; DCA, deoxycholic acid. [file Image4.jpeg]
